# The role of self-efficacy in specific fears

**DOI:** 10.1371/journal.pone.0283660

**Published:** 2023-03-24

**Authors:** Annalisa Lipp, Xiao Chi Zhang, Ekrem Dere, Armin Zlomuzica

**Affiliations:** 1 Department of Behavioral and Clinical Neuroscience, Ruhr-University Bochum, Bochum, Germany; 2 Department of Clinical Child and Adolescent Psychology, Ruhr-University Bochum, Bochum, Germany; University of Cadiz: Universidad de Cadiz, SPAIN

## Abstract

Low self-efficacy for threatening stimuli and situations has been proposed as an important etiological factor in the development and maintenance of specific phobias. The present study examined the relationships between general self-efficacy (GSE), specific self-efficacy (SSE) and specific fears in a representative sample (*n* = 717). While GSE was associated with higher self-reported fear and avoidance, SSE (e.g. SSE in the presence of animal-related fear) was more related to specific fears. SSE turned out to be a significant predictor of specific fear even after controlling for trait anxiety, age and gender. Interestingly, the association between SSE and specific fear differed across the different fear categories. Fear and avoidance of blood/injection/injuries showed the highest associations with SSE. In contrast, the association between natural environment-related fear and avoidance and GSE or SSE together was only modest. Exploratory analyses revealed a gender-specific effect on the strength of the association between SSE and specific fears. Women scored higher in animal-related fears and SSE. Our findings support the self-efficacy hypothesis of anxiety disorder development and provide a more detailed insight into the role of GSE and SSE in specific fears and phobias.

## Introduction

Specific phobias are characterized by intense and persistent fear reactions towards either specific stimuli and/or situations accompanied by strong avoidant behavior [1]. According to the common DSM classification system, five different categories or subtypes of specific phobias can be differentiated: animal, natural environment, blood/injection/injury (BII), situational and other phobias [1]. Significant differences in the prevalence [2], phenomenology [3] and pathophysiology [4] of specific fears and phobia subtypes have been reported.

An influential etiological approach to understand specific phobias and anxiety disorders is derived from Bandura’s self-efficacy theory [5]. It has been proposed that the degree of perceived or subjective self-efficacy is related to successful coping with threatening stimuli and fear-related stimuli and situations [5–8]. A distinction between domain-specific self-efficacy (SSE, [9]) and general self-efficacy (GSE) has been made. SSE designates the subjective estimation of coping capabilities in situations within a specific domain, e.g. in academic or social settings [10] but also in the presence of threatening situations and stimuli [6]. In contrast, GSE is not related to a specific task or domain but rather designates a general subjective confidence in one’s ability to overcome and master difficult and stressful situations irrespective of the domain. To this end, GSE has been related to a variety of psychological constructs, including optimism, self-regulation, self-esteem, depression and anxiety [11].

Higher GSE has been linked to low trait anxiety in a non-clinical sample [12], whereas lower GSE has been related to high trait anxiety [12] and higher social anxiety symptom severity in both patients and healthy controls [13]. Moreover, reduced GSE in adolescents seems to be associated with increased anxiety [14]. In adolescents, SSE scores are correlated with corresponding phobia types (e.g. social self-efficacy with social phobia), whereas GSE is correlated with all phobias, namely social phobia, separation anxiety and school phobia [14]. Furthermore, SSE has been shown to have connections with specific phobias such as agoraphobia [15], spider phobia [16] and height phobia [17] and predicts the anxiety levels within the phobia and the degree of avoidance behavior. Finally, perceived self-efficacy seems to be an important mechanism of cognitive behavioral therapy for phobias and anxiety disorders [9]. Increases in self-efficacy predict treatment-related decreases in symptoms and increases in functioning in patients with anxiety disorders [18]. Enhancing self-efficacy during exposure therapy can improve treatment outcomes in patients with anxiety disorders [19, 20].

However, the exact relationship between GSE and/or SSE and different specific fears and phobia subtypes has not been investigated so far. This is surprising, given that the pathophysiology of some specific fears, e.g. BII-related fears and phobias, can be distinguished from other specific fears and phobia subtypes [21]. Cognitive factors related to certain specific fears seem to be important for some fear subtypes, e.g. “fear of losing control” in BII fears and phobias [21]. However, cognitive factors underlying specific fears can also differ across different fear categories [22]. The same might apply to self-efficacy. Individual’s perceived belief of being able to exercise control over the occurrence of potentially harming events contributes to fear and avoidance. Self-efficacy, thus, is an important cognitive component of specific phobia. But does the role of GSE and SSE differ depending on specific phobia subtypes? This research question has not been investigated so far.

Lifetime prevalence rates of specific fears and phobias vary from 7.69% to 19.9% [23–26], whereby women (10% - 26.5%) show higher prevalence rates relative to men (7.3% - 12.4%, [25, 26]). Furthermore, sex-specific differences seem to exist among the different specific phobia subtypes: animal, situational and natural environment phobias have the highest prevalence among women (12.1%, 3.2–5.4%, 2–7.5% respectively, [25]). However, findings regarding gender differences in prevalence rates for BII phobia are rather inconclusive [23, 24]. Specific phobia comprises distinct heterogeneous disorders which are additionally influenced by gender [27]. The complexity between gender effects and variability of specific phobia subtypes has not been sufficiently considered in etiologic research [28–31]. Interestingly, there is some evidence suggesting that GSE varies across genders, with men scoring higher in GSE than women [32]. Likewise, gender differences in domain-specific self-efficacy have been reported, e.g. academic self-efficacy (higher in male students, [33]), social self-efficacy (higher in female adolescents, [34]) and computer self-efficacy (higher in male students, [35]). The current study, therefore, examined whether gender differences in self-efficacy levels are functionally related to gender differences in specific fear subtypes.

We performed an online survey in a representative sample including questionnaires measuring symptoms of four different specific fears (animal, natural environment, situational and BII), the corresponding SSE scales and the GSE scale. We predicted that there will be a negative association between specific fears and GSE and SSE and that the association might be weaker for GSE relative to SSE. We further expected different associations between GSE and SSE depending on the specific fear category. Further exploratory analyses were performed to examine the contribution of gender effects in the above-mentioned predicted associations. In a subsample of participants, the trait anxiety questionnaire [36] was used to control for a possible influence of trait anxiety on the sense of self-efficacy and specific fears as shown earlier [14]. Finally, we predicted that all SSE scores would maintain their correlation with specific fears even when controlling for trait anxiety.

## Methods

### Participants and procedure

The study was pre-registered (https://osf.io/n5pfe) and included a non-clinical sample of 717 participants and a subsample of 351 participants that received an additional questionnaire on trait anxiety [36]. Participants that indicated having a psychological disorder were excluded before filling out the questionnaires. We also excluded participants with incomplete responses and participants that took less than 9 minutes to complete the study from our sample (*n* = 357). Overall, the participants were aged between 18 and 69 and were predominantly female (80% female), which is representative of the gender distribution of phobias.

Participants were recruited via the psychology faculty’s website and advertisements on social media and were asked to take part in the study by clicking on a link. First, they received information about the study and had the chance to ask the researchers questions. Then, they gave their informed consent online and completed the questionnaires. As participation compensation, students received research hours and other participants had the chance of winning an Amazon gift card. All experimental procedures were approved by the local ethics committee of the Ruhr University Bochum and were carried out in accordance with the principles outlined by the Declaration of Helsinki.

### Questionnaires

The *GSE Scale* (GSE, [37]) contains ten items and measures how confident the person feels to overcome difficult situations based on their self-assessed competence. The GSE items are scored on a four-point scale with 1 = *not at all true*, 2 = *hardly true*, 3 = *moderately true and* 4 = *exactly true*. The total score is determined by summing up all item scores, which results in a score between 10 and 40, where the higher scores represent higher GSE.

SSE for each phobic fear was measured by the *Self-efficacy Questionnaire for Phobic Situations* (SEQ-SP, [38]). The original version of the SEQ-SP has been translated into German and translated back into English by a native speaker for validity control purposes. The questionnaire consisted of 13 items and was applicable to any phobic situations, including animal, natural environment, situational and BII phobia. The items described specific phobic situations for which the participants had to assess their self-efficacy expectations based on a five-point scale (1 = *really sure I couldn’t*, 5 = *really sure I could*).

The *Specific Phobia Questionnaire* (SPQ, [39]) was used to measure fear symptoms and interference with daily activities among the different phobic categories. We adjusted the questionnaire to measure avoidance instead of interference with daily activities because avoidance of feared objects/situations is an essential diagnostic criterion in specific phobias [1]. We, therefore, measured two indices of phobia: fear intensity and avoidance. The SPQ contains 43 items out of which we used 41 items to measure the four phobic categories in question, more precisely ten animal items (e.g., "spiders"), nine environmental items (e.g., "High, open places"), eight situational items (e.g., "Tunnels"), and 14 BII items (e.g., "blood donation"). The items were evaluated on a five-point scale from 1 = *no fear/avoidance* to 5 = *extreme fear/avoidance*. The cut-off value for SPQ fear scores to detect individuals with DSM-5-specific phobia types is 8 for the animal category, 15 for natural environment, 4 for situational and 20 for BII [39].

Trait anxiety was measured with 20 items of the *State-Trait Anxiety Inventory* (STAI, [36]). The items were measured on a four-point scale ranging from 1 = *hardly ever* to 4 = *always*. The total score of trait anxiety was calculated by summing up the scores (ranging from 20 to 80), whereby higher scores were indicative of higher trait anxiety.

### Statistical procedures

Statistical analyses were carried out with RStudio [40]. The correlational analyses for the relationships between GSE, SSE and specific fears were performed with Spearman correlations. We used Spearman correlations because most variables (except the specific self-efficacies) showed high kurtosis and Spearman correlation is the preferred test for heavy-tailed distributions [41]. For examining the role of self-efficacy and trait anxiety in fear intensity multiple linear regressions, with gender, age, trait anxiety, GSE and SSE as predictors, were conducted. Furthermore, we performed an exploratory analysis with multiple two-tailed independent samples t-tests to examine gender effects in GSE, SSE and their association with the different specific fears. Holm-Bonferroni correction was applied to all t-tests to counteract the potential problem of multiple comparisons. A result was considered significant when p-values smaller than 0.05 were obtained.

## Results

### Descriptive statistics

Demographic information and test results of the sample are shown in Table 1. A comparison of the specific fear scores (animal: *M* = 17.04, *SD* = 5.67; natural environment: *M* = 15.32, *SD* = 5.17; situational: *M* = 12.32, *SD* = 4.22; BII: *M* = 23.06, *SD* = 8.60) suggests that fear was more prevalent in situations related to animals and natural environment, whereas it was the lowest in situation-related fear. For SSE, the results show that animal-related SSE had the lowest scores while situation-related SSE had the highest scores.

**Table 1:** Descriptive statistics.

	Mean	Median	Std. Dev.
**Age**	25.30	23.00	7.12
**GSE**	29.21	30.00	4.68
**Trait Anxiety**	42.18	41.00	10.27
***Specific Fear***			
**Animal**	17.04	16.00	5.67
**Natural Environment**	15.32	14.00	5.17
**Situational**	12.324	11.00	4.22
**BII**	23.06	20.00	8.60
***Self-efficacy***			
**Animal**	40.58	40.00	11.62
**Natural Environment**	47.07	47.00	11.26
**Situational**	47.85	48.00	11.17
**BII**	46.20	46.00	11.35

*Notes*. Std. Dev. = Standard Deviation; Trait Anxiety was only measured in the subsample. Please note the numbers of items differed for the categories of specific fear.

### Relationships between GSE, SSE and specific fear

Spearman correlation analyses were used for all demographical data and questionnaire scores, namely gender, age, GSE, SSE and specific fear (see Table 2). The correlations between the different fear categories revealed that fear related to natural environment and situations correlated highly with each other (*r*(715) = .64, *p* < .001) and BII-related fear seemed to be more distinct as it only had moderate correlations with the other fear categories (see Table 2). Furthermore, GSE scores correlated negatively with all fear categories, among which situation-related fear correlated the highest (*r*(715) = -.35, *p* < .001) and BII-related SSE the lowest (*r*(715) = -.23, *p* < .001). This difference was confirmed through a significant result of a Fisher’s z-Test (*z* = 2.48, *p* = .007). This outcome implies that higher GSE scores are related to less fearfulness, although these relations seem to be weak. The analysis of data related to avoidance of phobic situations followed the same pattern. In particular, avoidance was highly correlated with specific fear (animal fear: *r*(715) = .81, *p* < .001; situational fear: *r*(715) = .77, *p* < .001; natural environment fear: *r*(715) = .81, *p* < .001; BII fear: *r*(715) = .80, *p* < .001) and showed the same pattern with respect to the association with GSE (animal avoidance: *r*(715) = -.23, *p* < .001, *z* = -.60, *p* = .27; situational avoidance: *r*(715) = -.30, *p* < .001, *z* = -1.06, *p* = .15; natural environment avoidance: *r*(715) = -.29, *p* < .001, *z* = -.21, *p* = .42; BII avoidance: *r*(715) = -.24, *p* < .001, *z* = -.20, *p* = .42) and SSE (animal avoidance: *r*(715) = -.53, *p* < .001, *z* = -.26, *p* = .40; situational avoidance: *r*(715) = -.39, *p* < .001, *z* = -.68, *p* = .25; natural environment avoidance: *r*(715) = -.47, *p* < .001, *z* = 0, *p* = .5; BII avoidance: *r*(715) = -.52, *p* < .001, *z* = .79, *p* = .21). The correlations between the different specific fears and SSE were higher than the correlations with GSE and actually followed an opposite pattern. Specifically, BII-related fear had the highest negative correlation with SSE (*r*(715) = -.55, *p* < .001), whereas situation-related fear showed the lowest correlation (*r*(715) = -.42, *p* < 0.001). This difference was supported by a significant result of a Fisher’s z-Test (*z* = 3.23, *p* = .001).

**Table 2:** Correlations table.

	1.	2.	3.	4.	5.	6.	7.	8.	9.	10.	11.
**1. Gender**	-										
**2. Age**	-.17***	-									
**3. GSE**	-.12**	.12***	-								
**4. SPQ-Animal**	.11**	-.04	-.26***	-							
**5. SPQ-Environment**	.09*	-.01	-.28***	.50***	-						
**6. SPQ-Situation**	.16***	-.03	-.35***	.40***	.64***	-					
**7. SPQ-BII**	.06	-.03	-.23***	.38***	.39***	.39***	-				
**8. Animal-SSE**	-.17***	.12**	.23***	-.52***	-.34***	-.27***	-.18***	-			
**9. Environment-SSE**	-.04	.02	.27***	-.30***	-.47***	-.36***	-.26***	.37***	-		
**10. Situation-SSE**	-.03	-.05	.23***	-.27***	-.35***	-.42***	-.26***	.34***	.46***	-	
**11. BII-SSE**	-.04	.01	.23***	-.21***	-.30***	-.30***	-.55***	.24***	.45***	.40***	-

Notes

*p < 0.05

**p < 0.01

***p<0.001

BII-SSE = BII-related SSE; Environment-SSE = natural environment-related SEE; Situational-SSE = situation-related SSE; Animal-SSE = animal-related SSE.

### Regression models for all specific fear categories

To approach the question of which factors have the highest predictive power for the specific fears, multiple linear regressions with the subsample were conducted (see Table 3). In the multiple linear regression model for animal-related fear, gender (*β* = -.05, *p* = .30), age (*β* = -.02, *p* = .73) and GSE (*β* = -.03, *p* = .67) were not significant predictors. The overall regression model was statistically significant (*R*^*2*^ = .23, *F*(5, 345) = 20.43, *p* < .001) and accounted for 23% of the variation in animal-related fear. SSE (*β* = -.39, *p* < .001) and trait anxiety (*β* = .18, *p* = .004) were significant predictors with SSE predicting the largest amount. Next, a regression analysis was performed to evaluate the prediction of natural environment-related fear. The analysis yielded three insignificant predictors, which were GSE (*β* = -.05, *p* = .43), age (*β* = -.01, *p* = .84) and gender (*β* = -.01, *p* = .85). Trait anxiety (*β* = .21, *p* = .001) and SSE (*β* = -.37, *p* < .001) significantly predicted environment-related fear and yielded a significant model, *R*^*2*^ = .24, *F*(5, 345) = 21.79, *p* < .001, that can explain 24% of the variance in natural environment -related fear with SSE explaining the largest amount. For situation-related fear, the analysis showed that all variables except gender (*β* = .03, *p* = .49) and age (*β* = .02, *p* = .74) significantly predicted situation-related fear and together explained 24% of the variance, *R*^*2*^ = .24, *F*(5, 345) = 22.11, *p* < .001. Among the predictors, SSE turned out to be the most influential (*β* = -.29, *p* < .001) and trait anxiety the least (*β* = .15, *p* = .01). Lastly, a linear regression model for BII-related fear was conducted which showed that gender (*β* = -.02, *p* = .75), age (*β* = -.02, *p* = .63), GSE (*β* = -.06, *p* = .34) and trait anxiety (*β* = .08, *p* = .18) did not predict BII-related fear. In total, the model explained 30% of the variance of BII-related fear (*R*^*2*^ = .30, *F*(5, 345) = 29.17, *p* < .001) with SSE being the only significant predictor (*β* = -.51, *p* < .001).

**Table 3:** Regression models for all specific fears.

Predictor variables	*β*	*β*	Fit
		95% CI	
***Animal***			
Gender	-.05	[-1.30, 1.20]	
Age	-.02	[-.09, .06]	
Trait Anxiety	.18**	[.11, .24]	
GSE	-.03	[-.17, .12]	
Animal-SSE	-.39***	[-.44, -.34]	
			*R*^*2*^ = .23***
***Natural Environment***			
Gender	-.01	[-1.02, 1.00]	
Age	-.01	[-.07, .05]	
Trait Anxiety	.21***	[.16, .27]	
GSE	-.05	[-.17, .07]	
Environment-SSE	-.37***	[-.41, -.33]	
			*R*^*2*^ = .24***
***Situational***			
Gender	.03	[-.85, .91]	
Age	.02	[-.04, .07]	
Trait Anxiety	.15*	[.10, 0.20]	
GSE	-.19**	[-.30, -.09]	
Situational-SSE	-.29***	[-.33, -.25]	
			*R*^*2*^ = .24***
***Blood-Injection-Injury***			
Gender	-.01	[-1.75, 1.72]	
Age	-.02	[-.12, .08]	
Trait Anxiety	.08	[-.01, .17]	
GSE	-.06	[-.26, .15]	
BII-SSE	-.51***	[-.57, -.44]	
			*R*^*2*^ = .30***

Notes

*p < .05

**p < .01

*** *p* < .001

BII-SSE = BII-related SSE; Environment-SSE = natural environment-related SSE; Situational-SSE = situation-related SSE; Animal-SSE = animal-related SSE.

### Exploratory analyses of gender differences

To explore possible gender differences in GSE, SSE and the association with the different fears, we performed multiple two-tailed independent samples t-tests. Animal- (*t*(710) = -2.65, *p* = 0.048) and situation-related fear (*t*(710) = -2.92, *p* = 0.028) were significantly higher in women than in men whereas no gender differences were found for the other specific fears (natural environment: *t*(710) = -1.66, *p* = 0.49; BII: *t*(710) = -1.43, *p* = 0.62). Animal-related SSE was higher in men than in women (*t*(710) = 4.54, *p* < 0.001), while there were no differences in the SSE for the other specific fears (natural environment: *t*(710) = 1.30, *p* = 0.62; situational: *t*(710) = 0.95, *p* = 0.62; BII: *t*(710) = 1.36, *p* = 0.62). Interestingly, a significant gender difference in GSE was found, with men scoring higher in GSE relative to women (*t*(710) = 3.60, *p* = 0.002).

## Discussion

Our results are in line with the general proposition of the self-efficacy concept of anxiety that the perceived ability to cope with threatening stimuli and situations contributes to specific fears and phobias [6]. Although GSE was related to specific fears, as previously reported for anxiety and negative affect [11], a relatively stronger association between SSE and specific fear and avoidance was found. GSE seems to be related to a broad range of personality factors (such as optimism or self-esteem, [11]), but all in a moderate range. Likewise, we could show that GSE is related to all different fear categories to a small significant extent, whereas SSE had moderate to high relations to its corresponding fear. This is in accordance with previous findings by Muris [14], who showed that SSE is most strongly related to specific types of anxiety disorders in adolescents. Our findings support the conclusion that this pattern also exists in adults and may be applied to specific fears. To further examine the relationships between SSE and specific fears, we have demonstrated that this relationship stays robust even when controlling for trait anxiety and gender. Interestingly, GSE lost its significance or was the smallest predictor for specific fear, while SSE was the strongest predictor for all specific fears. These findings support the assumption that SSE is more linked to specific fears than trait anxiety. Our results indicate that trait anxiety and self-efficacy may display unique connections to specific fears and phobias.

BII-related fear demonstrated the highest association with SSE compared to all other specific fears. At the same time, BII-related fear was not related to GSE after controlling for SSE. In contrast, natural environment-related fear showed the lowest overall connection with self-efficacy. These findings demonstrate that certain irrational cognitions and beliefs are more tied to certain phobic subtypes [22]. Thorpe & Salkovskis [22] for example showed that spider phobics exhibit higher levels of belief in the amount of harm than non-spider phobics and non-phobic individuals. In contrast to other phobia subtypes, BII-related phobia is associated with strong diphasic physiological reactions [21] which may result in fainting. Fear to faint in public situations is central to BII phobia [42] and thus differs from beliefs and concerns which are more characteristic of other phobia subtypes [22]. It is possible that BII-related fears, i.e. beliefs to have a decreased control over bodily reactions and a belief to be unable to exert control over strong physiological reactions (fainting, [21]) are linked to lower SSE. While this conclusion is speculative, our results nevertheless support the conclusion that the cognitive determinants (and thereby different associations with self-efficacy) related to different fears might vary.

Women are more affected by specific phobias than men (e.g. [43]). The most common forms of phobias in women are those related to animals and heights [44]. In line with this, we found that animal- and situation-related fear are higher in women relative to men. This gender effect, however, was not found for other specific fears. In accordance, gender effects were detected in animal-related SSE, whereas no gender differences were found for other specific fears. Gender effects in situation-related fear were not evident in situation-related SSE, probably because a combination of multiple factors predicts situation-related fear. However, the current findings suggest that gender effects in animal-related fears might be explained by gender differences in animal-related SSE. Animal phobias often have their main focus on feelings of disgust [3] and are generally associated with higher disgust sensitivity [45]. Women show higher levels of disgust sensitivity [46] and therefore might be more disgusted by animals such as spiders. High disgust reactions during animal encounters might cause a sense of inability to cope with these situations. Consequently, this low perceived self-efficacy might lead to avoidance of animals and higher levels of fear towards them [16].

The exact mechanisms by which cognitive determinants such as SSE contribute to specific fears and phobias remain elusive. Lower self-efficacy can have an effect on the individual’s capacity to learn from potentially threatening situations. We have shown that lower perceived self-efficacy is related to impaired discriminative fear learning [47], i.e. the ability to distinguish between safety and danger cues. Similarly, higher perceived self-efficacy can promote fear extinction learning [48], i.e. the ability to learn that a stimulus is no longer associated with potential harm. Fear learning and extinction constitute important mechanisms in the development of anxiety disorders [49]. Psychological treatments based on extinction learning and the promotion of self-efficacy are very effective in reducing fear and avoidance related to specific phobias (e.g. [19, 20]). Our findings support the conclusion that interventions aimed to alleviate fear and avoidance related to specific stimuli and situations should target self-efficacy as a central component.

The current study examined a non-clinical sample, which gives a good understanding of the relationship between specific fears and self-efficacy. However, a comparable study in a clinical sample with phobic participants is warranted. Furthermore, our results do not allow any causal explanations of the relationship between self-efficacy and specific fears and phobias. Decreased self-efficacy might potentiate fear and vice versa. Here, the use of experimental paradigms to increase or decrease perceived self-efficacy (see [19, 47, 48]) and examine the effect of self-efficacy interventions on fear and avoidance related to specific fears might be interesting.

In conclusion, our results indicate that SSE strongly contributes to fear and avoidance related to different fear categories. In support of the self-efficacy concept of anxiety, we showed that perceived inefficacy to cope with threatening situations might represent an etiological factor which contributes to the development and maintenance of specific fears and phobias. SSE seems to be especially relevant for certain types of fears, e.g. BII-related fears which are characterized by a decreased control over bodily reactions and/or belief of being unable to exert control over strong physiological reactions (fainting, [21]). A gender-dependent influence on animal-related fears and corresponding self-efficacy was also found. Our findings can help to understand and enhance psychological treatments based on learning and the promotion of self-efficacy in the context of phobias and anxiety disorders.

## Supporting information

S1 File Sub sample. (CSV)

S2 File Total sample. (CSV)
